# Life skills workshop impact: enhancing classroom adaptation for at-risk primary students in Chile

**DOI:** 10.3389/fpsyg.2026.1750734

**Published:** 2026-02-20

**Authors:** Alejandro Cuadra-Peralta, Constanza Beatriz Veloso-Besio, Camila Chamorro, Michelle Ibergarai-Pérez, Lorena Gallardo-Peralta

**Affiliations:** 1 Department of Psychology and Philosophy, Universidad de Tarapacá, Arica, Chile; 2 Universidad de Tarapacá, Arica, Chile; 3 National School Aid and Scholarship Board, Arica, Chile; 4 Department of Social Work, Universidad Complutense de Madrid, Madrid, Spain; 5 Department of Social Work and Social Services, Universidad Alberto Hurtado, Santiago, Chile

**Keywords:** child mental health, life skills program, school adjustment, school intervention, social-emotional learning, socio-emotional skills

## Abstract

**Background:**

Difficulties adjustment to school in childhood are one of the risk factors for developing mental health problems. When school adjustment problems arise, they tend to persist over time; therefore, early identification and intervention can help prevent these problems from worsening. In this vein, preventive strategies have been proposed based on developing socio-emotional skills that improve school adjustment. In Chile, school adjustment problems are addressed through preventive workshops based on social–emotional learning, which conforms to Life Skills Program 1.

**Objective:**

This research aimed to analyze the effectiveness of preventive workshops belonging to Life Skills Program 1 to improve adjustment to the classroom among Chilean primary school students.

**Materials and methods:**

The design of this research was a retrospective quasi-experimental type, with pre -and post-test measurements. The experimental condition consisted of 185 children in the 1st grade of primary school who were classified in the at-risk group in the *Teacher Observation of Classroom Adaptation-Revised*. These children received preventive workshops of the Life Skills Program 1. Secondary data were used for data analysis.

**Results:**

In an initial sample of 1.482 students, 185 were classified as at risk using the TOCA-RR instrument and received the intervention. After the intervention ended, 123 students were no longer at risk, while 62 remained at risk. Results evidenced that workshops were successful in 66.5% of the cases.

**Conclusion:**

The preventive workshops are effective in reducing school adjustment problems in at-risk children, which, if not treated in time, could trigger mental health problems in childhood that could extend into adulthood.

## Introduction

According to Leiva et al. (2020), one of the risk factors for developing mental health problems is adjustment difficulties to school. This is especially relevant given that adaptation to the school environment is one of the main challenges in child and adolescent development. When school adaptation is successful, psychosocial adjustment skills transfer to other contexts, and subjective well-being increases.

However, Leiva et al. (2020) have pointed out that when school adjustment issues arise—such as low motivation, lack of participation in classroom activities, negative attitudes toward teachers, and difficulties in adhering to rules and standards of behavior—they tend to persist over time. Therefore, early identification and intervention are crucial for addressing these problems promptly.

It is important to note that difficulties in school adjustment, particularly during the initial years of primary education, are linked to the emergence of various issues, including mental health and psychopathological problems, both in the short, medium, and long term [Ministry of Social Development and Family (MSDF), 2021].

Adaptive difficulties at school would be a consequence of limitations in children’s cognitive, emotional, and social resources, which would negatively affect the normal course of their development, causing mental health problems that would hinder children from being able to meet school expectations [Alcaíno, 2013; Ministry of Social Development and Family (MSDF), 2021]. To address these challenges, strategies have been proposed that focus on developing socio-emotional skills to enhance school adjustment (Leiva et al., 2020).

Social–emotional competence is a multidimensional construct that includes both intrapersonal and interpersonal skills which are essential for promoting positive adjustment and ensuring success in school. Intrapersonal skills are those necessary for individuals to function effectively on their own. These skills include realistic goal-setting, maintaining a positive mindset, self-control, emotion regulation, and coping strategies. In contrast, interpersonal skills are essential for successful interactions with others. Examples of these skills are active listening, effective communication, perspective-taking, negotiation, and social problem-solving (Domitrovich et al., 2017; Nakamichi et al., 2021; Pervez and Galea, 2024). These competencies are especially important for those children who are at risk due to economic disadvantage, minority status, or early emotional and behavioral problems (Payton et al., 2008).

Concerning the above, the socio-emotional learning process enables children and young people to acquire and effectively apply the knowledge, attitudes, and skills necessary to understand and regulate emotions, set and achieve positive goals, feel and show empathy for others, establish and maintain positive relationships, and make responsible decisions, among others (Domitrovich et al., 2017). Socio-emotional skills are predictive of major life outcomes like educational attainment, employment, earnings, health, and participation in crime (Belfi and Borghans, 2025; Sorrenti et al., 2025).

In this vein, research shows that the successful development of social–emotional skills is associated with greater well-being and improved academic performance. Conversely, the inability to cultivate these skills can lead to challenges on personal, social, and academic levels (Becker et al., 2014; Durlak et al., 2011; Fredrick et al., 2022; Hosokawa et al., 2024; Murano et al., 2020; Suldo et al., 2014; Weissberg and Greenberg, 1998).

Early social and emotional competence, which is connected to important life skills, is viewed as the foundation for healthy development. This competence is associated with long-term life outcomes, such as success in the job market, reduced rates of criminal behavior and drug abuse, and a lower risk of developing mental health issues later in life (Sorrenti et al., 2025; Wigelsworth et al., 2022).

In recent years, there has been an increasing recognition of the importance of learning socio-emotional skills alongside cognitive and academic skills within the educational system. Schools play a fundamental role in promoting these skills (Durlak et al., 2011). As a result, life skills have received considerable attention from educational policymakers, researchers, and educators worldwide over the last decade. These skills are viewed as essential for later life success and are a key aspect of quality education (Murphy-Graham and Cohen, 2022).

Research indicates that preventive socio-emotional learning programs in primary education significantly impact student development. These programs have shown positive effects through various beneficial outcomes (Wigelsworth et al., 2022). Specifically, high-quality and well-implemented social–emotional learning programs can enhance both intra- and interpersonal skills, leading to improved social and emotional abilities, better attitudes toward school, higher academic performance, and a reduction in mental health issues (Wigelsworth and Lendrum, 2016). Additionally, these programs contribute to better behavioral adjustment, resulting in increased prosocial behavior and a decrease in internalizing problems (Durlak et al., 2011). In a meta-analytic review, Ağirkan and Ergene (2022) examined the effect of social–emotional learning-based interventions, finding that social–emotional skills improved and externalizing and internalizing problems were significantly reduced.

The development of socio-emotional and problem-solving skills constitutes protective factors for mental health (Leiva et al., 2015b). Therefore, the need for the schools to be comprehensive, encompassing the support, education, and emotional development of children, is highlighted (Wigelsworth and Lendrum, 2016). In this vein, schools are crucial in promoting the comprehensive health of children, enhancing both their academic-cognitive development and socio-emotional evolution (Gimbert et al., 2023), with attention to their cultural context (El Mallah, 2022).

In accordance with national trends, school mental health in Chile has been addressed through preventive interventions aimed at supporting at-risk children and adolescents, specifically the Skills for Life Program ([SLP 1], Leiva et al., 2015b). This program focuses on developing socio-emotional competencies among participants. The SLP 1 is currently outlined in the National Mental Health Plan 2017–2025 of the Ministry of Health as a continuation of the program of the same name initiated in 1998, whose actions focus on improving the well-being and mental health of Chilean citizens [Ministry of Social Development and Family (MSDF), 2021]. The National School Aid and Scholarship Board (NSSB) is responsible for implementing this program, which has national coverage and, in collaboration with local governments, has been structured as a public response to mental health promotion and prevention (Leiva et al., 2015a).

The SLP is internationally recognized as the largest-scale school mental health program in Latin American countries and the fifth-largest mental health program worldwide. The SLP program is the most important school mental health program in Chile (Rojas-Andrade et al., 2025). This intervention program is based on the three-tiered model recommended by the World Health Organization (Murphy et al., 2017) and adheres to the guidelines set by the National Academy of Sciences of the USA to improve mental health for both girls and boys (Garfin et al., 2014).

The SLP is a permanent, multilevel, and multi-component school mental health public policy. It is designed to address mental health issues in educational communities and improve school performance by promoting positive environments and the development of processes of social–emotional learning (Rojas-Andrade and Leiva, 2019). This policy targets children and adolescents from kindergarten through secondary school (ages 4 to 18) and is structured into three phases. The SLP 1 covers students from the first to the fourth grade of primary school, between 6 and 9 years of age. SLP 2 considers students from the fifth to eighth grades of primary school, between 10 and 13 years old. The SLP 3 is applied to secondary students between 14 and 18 years of age. To date, the SLP has been carried out in 278 communes across the country, with coverage of 1.127.863 chilean students (Aranguren Zurita et al., 2022).

In general terms, the SLP outlines both short- and long-term objectives. The short-term goal is to improve academic performance, foster relationships within the school, and support students emotional well-being. The long-term objective focuses on improving the quality of life and preventing mental health damage that is associated with violent behavior, depression, and substance abuse (Rojas-Andrade and Leiva, 2018).

Concerning SLP 1, its purpose is “to favor a successful adaptation of both girls and boys during the first stage of school life through a school mental health intervention program for the development of social, cognitive, and affective competencies and skills inserted in the educational communities” (National School Aid and Scholarship Board, 2023, p. 1).

The SLP 1 preventive workshops are *targeted* in nature; that is, they intervene on specific risks identified by the *Teacher Observation Scale of Classroom Adaptation-Revised* (TOCA-RR). It should be noted that targeted prevention workshops address the person’s risk factors amd also intervene with their parents, teachers, and peers, as they may represent environmental conditions that influence the impact of the intervention. Thus, the preventive intervention of SLP 1 meets the characteristics of a *focused prevention strategy with a psychosocial approach* (Life Skills Manual, NSSB (Manual de Habilidades, JUNAEB), n.d.).

Among the theoretical bases underlying SLP 1 are the theories of *child development*, *social–emotional learning*, *problem behavior theory* (maladaptive), and *multiple intelligence theory* (Life Skills Manual, NSSB (Manual de Habilidades, JUNAEB), n.d.).

The definition of child mental health adopted by SLP 1 is grounded on *life course and social field theory* developed by Kellam and his collaborators. This theory emphasizes that during each stage of life, there are a few main social fields with specific demands for social tasks, one of which is the school (Alcaíno, 2013).

This theory proposes a two-dimensional construct of child mental health, with a social/external dimension called *social adaptation status* and an individual/internal dimension called *psychological well-being*. Social adaptation (linked to this research) refers to the appropriateness of an individual’s responses to specific social situations. Thus, social adaptation is the process of social task demands and behavioral responses. In addition to what is indicated, adapting or maladapting to earlier social task demands in specific social fields leads to later adaptation or maladaptation within the same field and in other social fields (Kellam et al., 2008).

In the case of children and young people, the school adaptation process unfolds within the school as a central social field. This process entails a tension between the demands of this context and students´ capacities to respond to social adaptation tasks [Ministry of Social Development and Family (MSDF), 2021]. According to Kellam et al. (2008), the first social task demanded of students is engaging in behavior that is in keeping with classroom rules, to which one maladaptive response is aggressive, disruptive behavior. In addition, there are other tasks of social demands, such as participating in social interactions with classmates and the teacher, not being too shy or withdrawn, paying attention, remaining focused, and learning academic subjects.

Regarding the above, Rojas-Andrade and Leiva (2018) reported the results of a nationwide study aimed at describing mental health difficulties among Chilean school students. This research was carried out from the perspective of 197 SLP 1 and SLP 2 implementers who were specialists in applying this type of intervention. Notably, these 197 professionals represented 75.59% of the various SLP projects executed in the country.

The findings revealed that 49.57% of the difficulties reported by the respondents were externalizing, including unspecified externalizing problems, attention-deficit/hyperactivity, aggressive behavior, disruptive behavior, and impulsivity. Additionally, 29.13% of the issues were internalizing, such as depression and anxiety, low self-esteem, among others; and 21.30% were school coexistence problems, including deficits in social skills, mistreatment, etc.

The authors emphasized that although externalizing behavioral problems represent one of the main demands in schools, they are also among the most manageable issues from a mental health perspective. This is due to the effectiveness of interventions in modifying externalizing trajectories when detected in time (Rojas-Andrade and Leiva, 2018).

Since the implementation of SLP 1, several studies have been conducted to evaluate its effectiveness. The initial validation studies revealed a significant reduction in maladaptive behaviors among children, including aggression, hyperactivity, and difficulties with concentration and attention. These behaviors can negatively impact learning, socialization, and adaptation to school. Notably, these improvements were also observed by the children’s natural evaluators, such as their parents and teachers (National School Aid and Scholarship Board, 2023).

As a result of SLP 1, there has been a productive line of national research that has facilitated its consolidation over the years. Today, SLP 1 is supported by a strong body of scientific evidence demonstrating its effectiveness. The findings indicate several positive outcomes, including the mitigation of the negative effects of exposure to natural disasters (Garfin et al., 2014); contributing to the relational repair of school actors; stimulating interactions and positive changes in the wellbeing of the educational community (George et al., 2012); increasing school performance (Delgado et al., 2006; Guzmán et al., 2011; Guzmán et al., 2015; Murphy et al., 2015); and decreasing school adjustment difficulties in students (Guzmán et al., 2015; Leiva et al., 2015a).

Although a significant body of research has emerged from SLP 1, further investigation is needed to evaluate the effectiveness of the preventive workshops to improve students’ adjustment to the classroom in the second year of primary. This is particularly important because such difficulty affects various spheres of the lives of at-risk children, as demonstrated throughout the Introduction.

Given the above, the objective of this research was to analyze the effectiveness of preventive workshops from the Life Skills Program 1 for girls and boys who experienced adjustment problems in the classroom, as measured by the TOCA-RR.

Based on this objective, the following hypothesis was established:

*H1:* Girls and boys who, in 1st grade, were classified as a risk group on the classroom adaptation scale (TOCA-RR), by carrying out preventive workshops in 2nd grade, will no longer belong to the risk group in 3rd grade.

## Materials and methods

### Participants

The original sample comprised 1,482 children, which constitute the total number of students in the 19 schools involved who were in first grade. Subsequently, the study was conducted with those students who presented psychosocial risk factors: 185 in first grade and 168 in third grade. More specifically, this research was divided into two phases. In the first phase, the total sample of 1.482 children was evaluated for psychosocial risk factors in first and third grade. The second phase considered only those children who showed psychosocial risk factors in either first or third grade. In this second phase, the sample consisted of 185 children who showed psychosocial risk factors in first grade, plus the 168 students who showed them in third grade.

The preventive intervention involved 185 students from the 1st Cycle of Basic Education (Primary), who obtained a risk score on the TOCA-RR. The children attended 19 subsidized schools in the city of Arica, in the Arica and Parinacota Region (Chile). In Chile, subsidized establishments are free. In Arica, according to Education Statistics (2018), p. 18), there are 78 schools of this type.

Of the 185 students, 80 were girls (43.4%) and 105 were boys (56.6%). In the Chilean educational system, the minimum age to enter the first grade of primary school is 6 years old as of March 31 (one month after the beginning of school). The mechanism for passing to the next grade is automatic promotion, so children in second grade are 7 years old, and in third grade, they are 8 years old.

### Procedure

This research was conducted in Chile, a middle-income country in South America. The city of Arica is located in the extreme north of Chile, quite far from the country, where the nearest city is 300 kilometers away; it has 189.644 inhabitants.

The design of this research was of a retrospective quasi-experimental type, with pre-and post-test measurements. This type of design is common; its main characteristic is that the participants self-select, or a provider does so on their behalf, to receive the treatment or intervention. It should be added that this type of design usually uses secondary data, such as databases already developed by those who implemented the intervention at first (Maciejewski, 2020).

Quasi-experimental designs are especially suited for evaluating the effectiveness of an intervention in real-world contexts (Handley et al., 2018), such as educational contexts. According to Gopalan et al. (2020), the growing demand for rigorous policy evaluation has contributed to the increased use of this type of design in educational research. Thus, quasi-experimental designs allow the evaluation of whether a program, policy, or intervention works as intended (Maciejewski, 2020).

To determine which students should receive SLP 1, a pre-intervention measurement was conducted using the TOCAR-RR while the girls and boys (*n* = 1.482) were in 1st grade at primary school. The criterion for inclusion in the workshops was based on the scores obtained by students in the TOCA-RR, as evaluated by their teachers. Therefore, students who received scores that categorized them as “at-risk” were invited to attend the preventive workshops.

Based on the results of the pre-test evaluation, the experimental group consisted of 185 students from 19 subsidized schools, to whom the SLP 1 was applied one year after the pre-test measurement; i.e., the intervention was administered when the children were in 2nd grade in 2018. The post-intervention measures were made one year after the preventive workshops were applied, when the children were in 3rd grade in 2019.

Secondary data corresponding to the NSSB database of SLP 1 was used. This database contained the results of the TOCA-RR questionnaire, corresponding to pre- and post-intervention measurements.

### Study variables

- Independent variable: Skills for Life Program 1.
- Dependent variable: school adjustment difficulties.

#### Skills for life program 1: practical and methodological aspects

The SLP 1 operates as follows: first, all 1st-grade primary students are evaluated (detection and prevention area) using the TOCA-RR. Students whose scores indicate psychosocial risk on the TOCA-RR are referred to the preventive workshops for next year. Secondly, the workshops are applied in the 2nd grade. Third, all students are re-evaluated in 3rd grade using the TOCA-RR (Life Skills Manual, NSSB (Manual de Habilidades, JUNAEB), n.d.).

These preventive workshops aim to reduce the impact of risk factors (aggressiveness, low cognitive performance, hyperactivity, and shyness) and enhance protective factors (communication, sociability, and expression of feelings) through the development of competencies and skills in children in an integrated and adaptive way to the school environment [Life Skills Manual, NSSB (Manual de Habilidades, JUNAEB), n.d.].

The SLP 1 preventive workshops contain 15 sessions in total: 10 for students, 3 for parents, and 2 for the head teacher. Ten sessions for the children were applied during the school day in groups of 6 to 10 students, and each session lasted 1.5 to 2 h.

The workshop modality applied to the students was group, closed, and mixed, and it was implemented at school. Workshops are run by the psychosocial team belonging to the program. This team is the primary responsibility of the psychologist or other similarly qualified professional, who must ensure the continuity of the preventive activity (Life Skills Manual, NSSB (Manual de Habilidades, JUNAEB), n.d.).

It should be added that the workshops were provided by the Chilean Ministry of Education. The workshops had a strong practical component, so it was not necessary for the children to be able to read or write. The majority of students can read by first grade, and the workshops are held in second grade. The instrument was completed by the teachers (TOCA-RR). It is important to remember that teachers play a crucial role in mental health within the school (Rojas-Andrade et al., 2024). The trainers were psychologists who, in turn, received specific training on how to conduct the workshops with the children. They were also supervised weekly by the lead author of this article.

The SLP 1, applied to the children, is structured into phases, objectives, and activities (Table 1). The intervention consists of five phases: (1) group integration; (2) recognition of norms and limits; (3) identification and expression of emotions; (4) conflict resolution; and (5) the closing phase, in which the integration of experiences and the evaluation of the workshop by the group are worked on. It should be added that the sessions aimed at children are grouped into the five phases mentioned (Leiva et al., 2015a; Life Skills Manual, NSSB (Manual de Habilidades, JUNAEB), n.d.).

**Table 1:** Phases, specific objectives, and activities of the SLP 1.

Phases	Specific objectives	Activities
1. Group integration	1. To establish the conditions to form a preventive group workshop with girls and boys, emphasizing initial recognition and group integration.	1. Who are we?
2. Recognition of norms and limits	2. To encourage children to know, accept, and love themselves.	2. Who am I, and what am I like?
3. Identification and expression of emotions	3. To encourage children’s independence, responsibility, and self-care.	3. How am I like?
4. Conflict resolution	4. To promote self-acceptance and acceptance of others.	4. I am good at…
5. Closing	5. To recognize their own and others’ feelings.	5. Respecting each other
	6. To express and share feelings with others.	6. I love and take care of myself
	7. To stimulate the values of solidarity, coexistence, and cooperation in the children.	7. Responsibility
	8. To develop self-control strategies.	8. What scares me? What do I do when I’m scared?
	9. To generate alternatives for conflict resolution.	9. My feelings
	10. To integrate the work done by the group during the workshop.	10. Playing with each other
	11. To recognize and project personal goals based on the experiences of the workshop.	11. Creating together
		12. Stop before acting
		13. Solving problems
		14. Saying goodbye

The objectives are: 1. To establish the conditions to form a preventive group workshop with girls and boys, emphasizing initial recognition and group integration. 2. To encourage children to know, accept, and love themselves. 3. To encourage children’s independence, responsibility, and self-care. 4. To promote self-acceptance and acceptance of others. 5. To recognize their own and others’ feelings. 6. To express and share feelings with others. 7. To stimulate the values of solidarity, coexistence, and cooperation in the children. 8. To develop self-control strategies. 9. To generate alternatives for conflict resolution. 10. To integrate the work done by the group during the workshop. 11. To recognize and project personal goals based on the experiences of the workshop [Life Skills Manual, NSSB (Manual de Habilidades, JUNAEB), n.d.].

The activities carried out during the workshops include: 1. Who are we?; 2. Who am I, and what am I like? 3. How am I like?; 4. I am good at…; 5. Respecting each other; 6. I love and take care of myself; 7. Responsibility; 8. What scares me? What do I do when I’m scared?; 9. My feelings; 10. Playing with each other; 11. Creating together; 12. Stop before acting; 13. Solving problems; 14. To say goodbye [Life Skills Manual, NSSB (Manual de Habilidades, JUNAEB), n.d.].

### Instrument

*Teacher Observation of Classroom Adaptation-R-Revised* (TOCA-RR), developed by Sheppard Kellam and collaborators at Johns Hopkins University and the American Institute for Research. This instrument is based on Kellam’s theoretical model of mental health (George et al., 2004), described previously in the Introduction section.

TOCA-RR measures school adjustment difficulties and identifies risk factors and maladaptive behaviors associated with mental health problems and with a greater probability of presenting psychiatric disorders and risky behaviors during adolescence (Kellam et al., 2008; Leiva et al., 2015a). This instrument, specifically, provides information about the student’s behavior in the classroom. The teacher answers it in the context of a structured interview conducted by trained interviewers; that is, the teacher is the one who evaluates the student’s school adaptation.

The TOCA-RR consists of two sections: (1) a 31-item structured interview on children’s behavior, and (2) overall ratings: the teacher rates, on a scale from 1 (*excellent*) to 6 (*failure*), the child’s performance as a student and their behavior in class.

The 31 items are grouped into four dimensions: (1) *Aggressiveness and hyperactivity* correspond to the risk factors of aggressiveness, disobedience, and hyperactivity. This dimension involves the presence of levels of aggressiveness above expectations and difficulties in behavioral regulation. These children frequently harm or hurt others, start fights with their peers, are disobedient, react badly to failure, and have difficulty staying calm. (2) *Cognition and learning* correspond to the risk factors of concentration problems and low motivation to learn. This dimension indicates the presence of difficulties in concentration and low motivation towards learning. (3) *Social contact* corresponds to the risk factor of shy behavior. This dimension involves the existence of difficulties in initiating and sustaining social relationships. These children are shy and unfriendly and have problems maintaining social relationships with their peers. (4) *Emotional maturity* corresponds to the risk factor of emotional immaturity. This implies dependence on their teachers or peers to achieve their social tasks. These children constantly need the teacher’s attention and cling to their classmates (SLP 1 Detection Unit, 2023).

Below are examples of items for each factor, the number of items, and their internal consistency: *aggressiveness and hyperactivity*: “start fights with classmates”; 11 items, *α*: 0.92. *Cognition and learning*: “completes assignments”; 9 items, α: 0.87. *Social contact*: “is sociable/interacts with classmates”; 6 items, α: 0.90. *Emotional maturity*: “clings to the teacher”; 5 items, α: 0.81 (SLP 1 Detection Unit, 2023).

It should be mentioned that the TOCAR-RR has solid theoretical foundations (life course/social field theory) and empirical evidence that guarantees its adequate technical quality (validity properties). These characteristics enable it to detect risk factors in schoolchildren and to evaluate the effectiveness of preventive workshops of the LSP 1 (George et al., 2004; Guzmán et al., 2015; Leiva et al., 2015a; SLP 1 Detection Unit, 2023).

According to George et al. (2004), several research teams have adapted and validated the questionnaire in Chile, which facilitates its use in the Chilean context.

### Analytical strategy

For data analysis, the SPSS program was used. To statistically contrast the study hypothesis (*Girls and boys who in 1st grade were classified as a risk group on the classroom adaptation scale (TOCA-RR), by carrying out preventive workshops in 2nd grade, will cease to belong to the risk group in 3rd grade*), the McNemar test was applied. The statistical significance level established was 0.05.

### Ethical considerations

This research was approved by the Ethics Committee of the Universidad de Tarapacá (Chile).

The National School Aid and Scholarship Board authorized the use of the database.

The results and conclusions presented in this research are the exclusive responsibility of the authors.

## Results

A total of 1.482 first-grade students were assessed, of whom 185 were identified as being at psychosocial risk and 1.297 as not. Those students identified as being at risk attended preventative workshops during their second year of primary school. Subsequently, in their third year of primary school, they were assessed again, and 168 of them were identified as being at psychosocial risk. Focusing solely on students at psychosocial risk in first and third grade, the results presented in Table 2 were obtained.

**Table 2:** Psychosocial risk assessment using the TOCA-RR.

Assessment	Students at psychosocial risk
1st assessment in 1st year	185
2nd assessment in 3rd year	168

When comparing the first (1st grade) assessment with the 3rd grade assessment, the differences are not significant. However, when evaluating only the students who participated in the preventative workshops, that is, the 185 children assessed as being at psychosocial risk in the 1st grade, the results are very different, as shown in Table 3.

**Table 3:** Students with and without risk after the preventive workshop.

Students after the prevention workshop	*n*	Percentages
Students at risk after the prevention workshop	62	33.5%
Students not at risk after the prevention workshop	123	66.5%

The apparent contradiction in the figures is because a total of 106 students, who in the 1st year were not at risk and, therefore, did not attend the preventive workshops, in the 3rd year did show psychosocial risk in the instrument.

Table 3 shows a first approach to the effectiveness of the preventive workshops, comparing the results of the TOCA-RR instrument in 1st and 3rd year of primary school, of the boys and girls who attended preventive workshops.

A total of 185 children were identified as being at psychosocial risk in the first year of primary school and 168 in the third year. However, of the total number of children at psychosocial risk in the third year, it is noteworthy that 106 of them did not participate in preventive workshops in the second year, as they were outside the cutoff point in the TOCA-RR instrument when they were in the first year of primary school.

**Figure 1:**
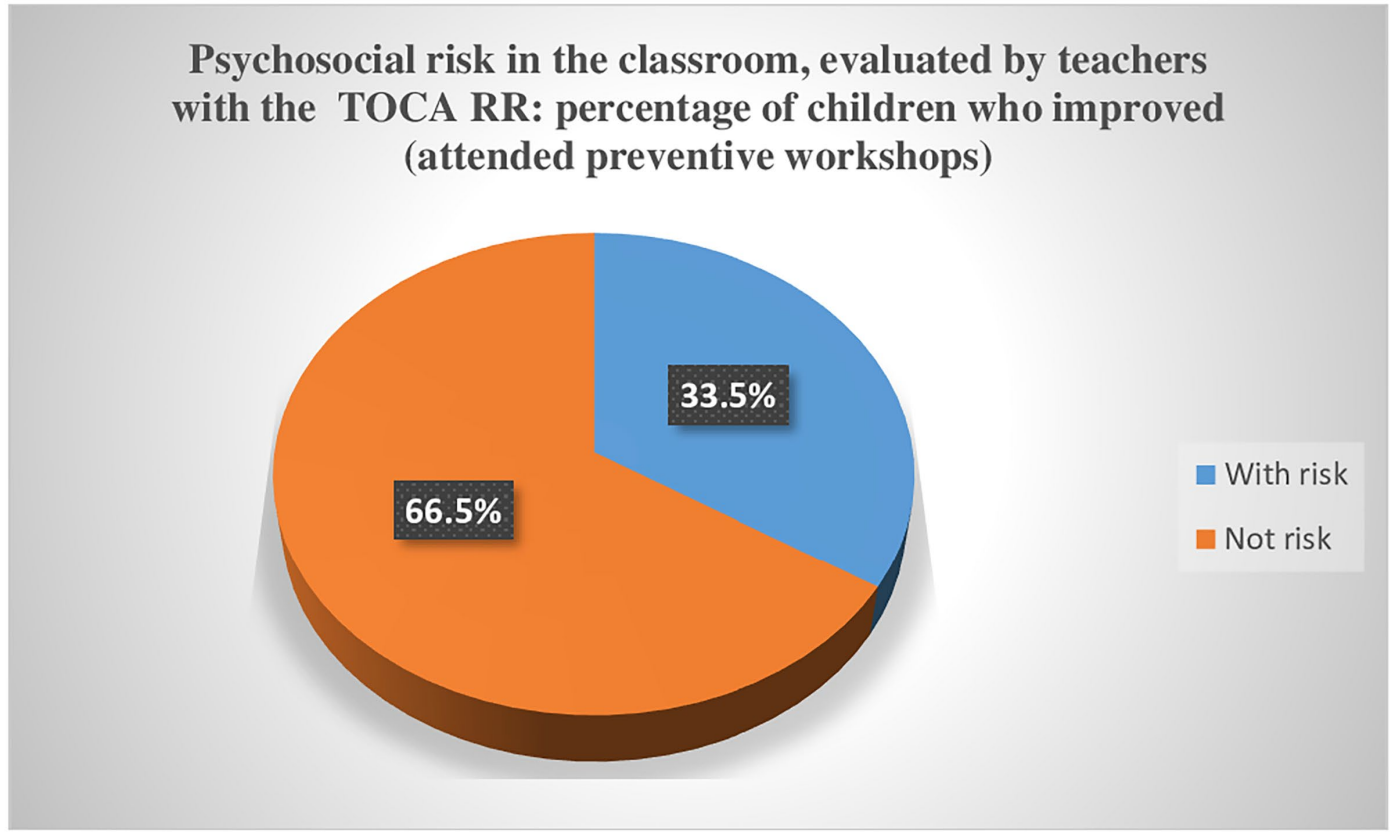
Effectiveness of preventive workshop.

When reviewing the data on boys and girls identified as having psychosocial risks in the 1st year of primary school—who subsequently attended preventive workshops in the 2nd year—it was found that 123 of these children no longer belonged to the risk group when re-evaluated in the 3rd year. This represents a 66.5% reduction of the total number of children initially identified as at risk (Figure 1).

Additionally, McNemar’s test was used, a non-parametric statistical test for paired (dependent) nominal data that compares differences in matched case–control studies. It is based on a 2×2 contingency table. This test is equivalent to the chi-square test for related samples (Tables 4, 5).

**Table 4:** 2×2 contingency table in the McNemar test.

		Risk in 3rd year		Total
		With risk	Without risk	
Risk in 1 year	With risk	62	123	185
	Without risk	106	0	106

**Table 5:** Hypothesis testing summary.

Null hypothesis	Statistical test	Significance	Decision
Preventive workshops have no effect on psychosocial risk	McNemar test for related samples	0.000	Reject the null hypothesis

As shown, among the children who attended the preventive workshops, the number of risk cases decreased from 185 to 62 (Table 4). This indicates a success rate of 66.5% and a significance level of 0.000 in the McNemar test.

## Discussion

This research aimed to analyze the effectiveness of preventive workshops from Life Skills Program 1 for children experiencing adaptation problems in the classroom, as measured by the TOCA-RR. Specifically, we tested the hypothesis that children who were classified as being in the risk group on the classroom adaptation scale (TOCA-RR) in 1st grade, and who participated in preventive workshops in 2nd grade, would no longer be classified as part of the risk group by 3rd grade.

The results indicated that out of the 185 children identified as at risk, 123 no longer fell into this category one year after participating in the preventive workshops. This indicates a success rate of 66.5% for the workshops. Therefore, it can be concluded that there is a statistically significant association between attending these workshops in second grade and no longer being at risk by third grade.

These findings align with the results of Guzmán et al. (2015) and Leiva et al. (2015a), who observed a reduction in school adjustment difficulties among Chilean students after implementing SLP 1. This information is significant, especially given that externalizing school adjustment problems are one of the primary concerns in Chilean schools. Fortunately, from a school mental health perspective, these issues are among the most solvable when detected early (Rojas-Andrade and Leiva, 2018).

It should be taken into account that, in this research, the results of the application of SLP 1 correspond to children between 6 and 9 years old who presented school adjustment problems, considered a risk factor for developing mental health problems (Leiva et al., 2020). We mention this since, in Chile, anxiety and disruptive disorders are the most frequent, with the highest prevalence in girls and boys between 4 and 11 years of age [27.8%, Ministry of Social Development and Family (MSDF), 2021].

It is important to highlight that out of the 1.297 students who were not initially classified as at-risk and did not participate in the preventive workshops, 106 of them regressed and became at-risk after one year. This finding warrants further investigation to identify potential causes for this regression.

### Limitations

One limitation is the lack of qualitative data capturing the perceptions of the parents and children who participated in the workshops.

It is important to emphasize the need for in-depth studies of both the children who regressed and those who did not achieve success after participating in the workshops.

As a limitation, it is important to recognize that quasi-experimental designs are the simplest of all to ensure internal validity (Handley et al., 2018). Therefore, the attribution of causality must be done with caution.

Another methodological limitation was that the design used in this research was retrospective (with secondary data). Thus, it wasn’t possible to identify and measure other variables, such as non-equivalent control variables, to optimize internal validity (Maciejewski, 2020). When using secondary data, we are aware of this type of limitation, so it should be made explicit.

Despite its limitations, this design allows for assessing whether an intervention has a positive or negative effect.

It is worth noting that experts have focused on refining quasi-experimental designs and have established guidelines to enhance these methods, ultimately improving the quality of empirical evidence (Handley et al., 2018). In consequence, the systematic application of this knowledge to the development of public policy-based interventions (and other types of interventions) should be the standard practice.

### Contributions

As a first contribution, this study contributes to enriching a line of research that receives increasing attention from the scientific community and other relevant actors, specifically the line of research on preventive mental health programs among children in low- and middle-income countries, such as Chile (Murphy-Graham and Cohen, 2022). In this line, SLP 1 directly intervenes in mental health risk factors in Chilean students, such as school adjustment difficulties.

As a second contribution, this research also highlighted the importance of identifying and monitoring a specific group of students. These are students who were not initially considered at risk and thus did not participate in preventive workshops, but were later classified as at risk. To address this issue, it would be beneficial to implement a systematic intermediate follow-up process. This approach could serve as an effective mechanism for detecting early signs of difficulties in school adaptation.

### Practical recommendations

We recommend implementing screening and preventive treatment for situations that affect children who are not at risk in the first year but become at-risk group in the third year. For example, those children who were not classified in the risk group by the TOCA-RR could experience family difficulties and not receive intervention due to their scores. It is worth considering that 106 children who were not at risk in the first year were in the risk group in the third year. We also recommend evaluating how close these children were to the cut-off point, since they may have been very close to being classified in the risk group; if so, less intensive preventive workshops can be provided.

### Suggestions for future research

Future research should focus on analyzing the characteristics of the group that attended the workshops but did not improve their risk status. Additionally, it would be beneficial to investigate the characteristics of the children who were not considered at risk in their first year but became at risk by their third year. These suggestions are crucial as the MSDF conducts a follow-up evaluation and monitoring process for SLP 1 to implement necessary reforms and improvements to the program. Therefore, these recommendations would provide valuable information for the follow-up evaluation and help optimize the preventive workshops.

As a methodological suggestion, we recommend measuring at least one non-equivalent outcome variable, i.e., a variable that does not change in response to the treatment of interest, also known as a falsification test or negative control results, to increase the internal validity of the quasi-experiment. In this line of thinking, the emphasis on evidence-based policies should translate into an interest in considering the methodological recommendations indicated by experts when designing these interventions. Thus, education researchers could make further progress in building a high-quality evidence base to refine the design of interventions based on public policies and, consequently, to optimize the precision in explaining the results obtained from them (Gopalan et al., 2020).

## Conclusion

In conclusion, the preventive workshops of SLP 1 can effectively reduce school adjustment problems in at-risk children, which, if not treated in time, can trigger mental health problems in childhood and extend into adulthood.

Finally, through the systematic evaluation of public policies designed to improve variables associated with school mental health, we hope to develop a more just and egalitarian society for those who need it most.

## Data Availability

The raw data supporting the conclusions of this article will be made available by the authors, without undue reservation.
